# The historical process of the masonry city walls construction in China during 1^st^ to 17^th^ centuries AD

**DOI:** 10.1371/journal.pone.0214119

**Published:** 2019-03-22

**Authors:** Qiaofeng Xue, Xiaobin Jin, Yinong Cheng, Xuhong Yang, Xin Jia, Yinkang Zhou

**Affiliations:** 1 School of Geography and Oceanographic Science, Nanjing University, Nanjing, Jiangsu Province, China; 2 Natural Resource Research Centre of Nanjing University, Nanjing, Jiangsu Province, China; 3 School of History and Archives, Yunnan University, Kunming, Yunnan Province, China; Gebze Teknik Universitesi, TURKEY

## Abstract

Masonry city walls were common defense facilities in the cities of the Eurasian before the industrial revolution. However, they were not widespread in China until the Ming Dynasty (1368–1644). Limited in research methods, previous studies failed to make convincing arguments on this phenomenon. We collected, organized and analyzed relevant historical documents to reconstruct the spatio-temporal process of the construction of masonry walls from 1^st^ to 17^th^ century in China. We conducted a time series analysis primarily based on factors such as wars, garrisons, economy, and natural disasters. Analysis of the correlation among the construction of masonry walls and these factors provides insights into this process. From the 1^st^ to 14^th^ century, only 125 masonry city walls were built in China and the annual average number was below 0.1. While in the Ming Dynasty, a total of 1,493 masonry walls were built, with an annual average of 5.41. The construction activities in 1368–1456 spread throughout the country, but mainly appeared in the high-grade administrative cities and garrisons, as a result of the planned implementation of the central government. The construction activities in 1457–1644 had corresponding cluster areas during different periods, mainly at county-level. We found that the wall construction was stimulated by external factors such as wars and disasters. We believe that the mass construction of masonry walls in the Ming Dynasty is a phenomenon of cultural diffusion. The central government plan, the complex interactions between local governments and community, and the stimulation of external factors worked together to contribute to the diffusion of masonry city walls in the Ming Dynasty.

## Introduction

Defense facilities including entrenchments, barriers, forts, and especially city walls, have become essential components for many ancient cities on the Eurasian continent before the industrial revolutions [1]. City walls were most frequently built by soil, rammed earth, bricks, stones, and even woods. Among these building materials, the use of masonry has a long history. Around 3,000 BC to 1,750 BC, cities of the Harappan civilization in the Indus Valley, such as Harappan and Moenjo-Daro, had used fired bricks to cover the city walls [2]. Such fired brick surface also appeared in the UR city of the UR III Dynasty from 2,100 BC to 1,900 BC [3]. Early in the 4^th^ century BC, Rome used 60cm-length bricks on city walls [4], and the more spectacular application of bricks were the Aurelian walls of Rome in 217 AD. Further, by the time of the Roman Empire (27 BC-330 AD), the masonry city walls had been widespread in Europe and the Middle East [5].

City walls were commonly seen in most ancient Chinese cities, and 14 city walls during the Ming and Qing Dynasties have been listed in the UNESCO World Heritage Tentative List [6]. However, compared to other major civilizations in the ancient world, masonry city walls appeared later in China, and even had not been popularized for a long time after their appearance. The Chinese began to burn bricks as early as on the Warring States period (453 BC-221 BC), but the primary use was in building tombs and rarely for ground buildings. The main construction approach of Chinese masonry city walls in the historical periods was to wrap bricks and stones on the surface of the earth walls [7], and it first appeared in the Eastern Han Dynasty (25 AD-220 AD) [8–9]. But in the Northern and Southern Dynasties (221–617), only a few masonry city walls were built [10]. From the Tang to the Yuan Dynasty (618–1367), the construction of masonry city walls gradually increased and concentrated in the south of China [11–12]. During this period, the traditional approach was to use soil to build rammed pounded earth walls, including the walls of capitals like Chang’an, Luoyang, and Dadu (Khanbaliq). A dramatic change occurred during the Ming Dynasty (1368–1644) while masonry city walls suddenly began to diffuse in China [13], and since then almost all Chinese cities had built masonry walls.

Historians and archaeologists have studied the reasons behind the mass construction of masonry walls in Ming Dynasty, and the general speculation was nothing more than the popularity of firearms, the national strength, the flood prevention and the building technology development during that period [14–17]. However, most of these arguments were based on limited examples of building city walls and simply attributed the cause to some ad hoc countermeasures against an issue. These inadequate analyses may not provide convincing explanations for the long-term and large-scale events. The diffusion of masonry walls in ancient China involved a huge number of cites and the process was very complicated, so it is essential to sort out its spatio-temporal process to improve understanding.

Ancient local chronicles of China [*Difangzhi*] had documented the events of building city walls with detailed construction records (e.g., time, scale, materials) in the volumes of “city walls and moats”. Thanks to these historical sources and current researches, we were able to quantitatively restore the temporal and spatial construction process of masonry city walls at the county level. In this study, we first collected and compiled these local chronicles that described the construction of masonry city walls. Then we reconstructed the spatio-temporal process of the events throughout China from the Eastern Han Dynasty to the late Ming Dynasty using a geospatial analysis software (i.e., ArcGIS 10.0) [18]. We built a time series model using factors (e.g., wars, garrison [Wei-suo] setting and abandonment, economy and disasters) that were identified by previous studies. Our goal was to explore the underlying causes of the popularity of masonry walls in the Ming Dynasty. Finally, we analyzed the mechanism of the events by incorporating cultural diffusion concept. Our research offers new insights for understanding the diffusion of masonry city walls in the Chinese history.

## Data and methods

### Location and construction time of masonry city walls

The research mainly included local administrative cities and cities with administrative functions (e.g., the guardian cities of the Ming Dynasty) from the Eastern Han Dynasty to the Ming Dynasty (25 AD-1644 AD); towns, military forts, or private castles were excluded. The location data for these cities were retrieved from the *General History of Chinese Administrative Divisions* [19] and the *Historical Atlas of Chinese* [20]. To reconstruct the spatio-temporal construction process of the masonry walls, we obtained the location and construction time of the city walls mainly from the local chronicle records compiled by the *Complete Collection of Pictures and Books of Old and New Times–Territory Canon*, *City walls and Moats Citation volume*. This giant book was compiled in 1728 and recorded the construction details of the city walls and moats (e.g., construction time and scale, repair and rebuilt) throughout China before the mid-Qing Dynasty (the18^th^ century). From a recently compiled version [21], we extracted 1,223 records (75.59% of the total data) of building masonry walls from the Eastern Han Dynasty to the end of the Ming Dynasty. However, the material did not document the following types of city walls: (1) the city walls of the abolished guardian cities of the Ming Dynasty, especially in the coastal areas and Guizhou Province, (2) the masonry walls built in the pre-Ming Dynasty, and (3) the city walls originally built as masonry walls. To supplement the missing data, we also retrieved data from other sources: (1) 130 records (8.03%) from the provincial chronicles compiled in the *Complete Library in Four Divisions*, (2) 60 records (3.71%) from the *Integration of Local Chronicles of Great Qing Dynasty*, (3) 70 records (4.33%) from the *Tianyige Ming Dynasty Local Chronicles Selection* and the *Tianyige Ming Dynasty Local Chronicles Sequel*, (4) 22 records (1.36%) from relevant military books of Ming Dynasty, (5) 35 records (2.16%) from the local chronicles of the Song, Yuan, Qing Dynasties and the Republic of China, and (6) 78 (4.82%) from the archaeological reports, new local chronicles and other current studies. This resulted in a total of 1,618 records of masonry walls from the Eastern Han to the Ming Dynasty. We converted all the time records into the year of the AD, and replaced the time-blurred records with the typical construction year of the masonry walls.

### War factors

We selected potential factors (e.g., wars, garrisons setting and abolishing, economy, and disasters) as mentioned in previous literature and analyzed their relationships with the diffusion of the masonry city walls in the Ming Dynasty.

Previous studies found that the diffusion of masonry walls in the Ming Dynasty were mainly associated with the popularity of firearms during war time [14–16]. Since it was difficult to distinguish use of firearms (e.g., quantity and degree of use) during each war in the Ming Dynasty. We chose the intensity of wars as a proxy of the use of firearms. We collected 650 records of wars during the Ming Dynasty from the *Chronology of Wars in China* [22]. This book compiled a comprehensive record of the wars from the 8^th^ century BC to the 20^th^ century based on the official record of the Ming and Qing dynasties, and summarized the cause, process, and the outcome for each war. To compare the wars of different scale, we used the “county time” (The year when a county was affected by a war) as a statistical unit to count the intensity of each war [23]. We divided 650 wars with different geographical scale into two categories: “foreign wars” and “internal wars”. The foreign wars mainly referred to wars in the frontier or coastal areas with the invasion of Mongolia in the north, Japanese pirates on the coast, and the Jurchen in Liaodong during the end of the Ming Dynasty. The internal wars mainly included the rebellion of the peasants, seigniors or troops in the inland.

### The garrison system

As the most basic military system in the Ming Dynasty, the garrison system served the city where they were located. Its set-up, abandonment, and relocation were closely related to wartime activities [24]. The garrison system of the Ming Dynasty also played a territorial management function. They were divided into “self-owned land garrisons” and “not own land garrisons” depending on the ownership or management of land. The “self-owned land garrisons” had no local governments accompanied and defended the cities and surrounding areas, while the “not own land garrisons” defended important local cities, but with no administrative power [25]. Studies found that the construction of the city walls during the early Ming Dynasty was clustered in the cities where the garrisons were set [26]. This finding indicates that the construction of masonry walls may be associated with the establishment of garrisons in the Ming Dynasty. Therefore, we collected 935 records of garrisons’ set-up and abandonment from the *Administrative Evolution Comprehensive Table in Ming Dynasty* [27] and the *General History of Chinese Administrative Divisions (Ming Dynasty)* [28], and used the annual net increase (the number of new set-up garrisons minus the number of abandonment garrisons) as the number of changes on the garrisons during the Ming Dynasty.

### Economic factors

As a public work, the cost of building the city wall was theoretically borne by the government. Previous research found that the project cost (per unit) of brick city walls in the Qing Dynasty was about 1.7 times that of the stone walls and 5 times that of the earth wall [29]. In the middle of the Ming Dynasty, to build a small city wall with a circumference of 3,000 meters would cost about 1,000 to 1,500 kilograms of silver. Bigger walls cost more [30]. The higher cost of masonry walls required stronger economic basis. However, in addition to water conservancy projects, there was no fixed budget for public works among the funds of local governments in the Ming Dynasty. Its financial expenditure arrangement depended on the local situation, and there was no consistent regulation. Due to the unstable financial expenditure of governments on public works, they were often just conveners, and local gentlemen and common people undertook more works [31]. As a result the actual cost of city walls construction may be 30% or even more than 50% higher than the official figures [30]. Nevertheless, the governments’ tax revenue was still an important economic basis to ensure the construction of city walls, and to a certain extent it could reflect the general situation of the economy, so it could be discussed as an influencing factor. The tax laws of the Tang Dynasties were followed in the Ming Dynasty, and agricultural tax was the main fiscal revenue of the government. We used the major financial revenue (i.e., rice and wheat income) of the central government as a proxy to the economic condition in the Ming Dynasty [32]. During 1368–1644, 137 years (49.64%) were recorded and 139 years (50.36%) were unrecorded. Among them, annual data during 1402–1522 was complete; 1522–1626 had data documented every 6.5 years; 1381–1391 for every 10 years. We interpolated the missing values of data from 1522–1626 and 1381–1391 using the averaged value of the previous and the following data. This process resulted in economic data of the rice and wheat income of the central government between 1381–1392 and 1402–1626 for 234 years (83.78%) in the Ming Dynasty.

### Disaster factors

Historically, the lack of food production may lead to famine and even induce peasant uprisings [33]. The construction of masonry walls might prevent the country from further deterioration and associate with the occurrence of disasters. We collected 2,846 disaster records of the Ming Dynasty from the *Disaster History in China (Ming Dynasty)* [34]. This book demonstrated the profiles (e.g., types and locations) of disasters in various forms based on a comprehensive study of natural disasters and disaster relief systems in the Ming Dynasty. Considering the various scales of the disasters, we used the “county time” (if one year a county was affected by the disaster, one “county time” was counted) as a general statistical unit to represent the intensity of each disaster. In addition, some studies aforementioned suggested that the diffusion of masonry walls in the Ming Dynasty was related to the prevention of flooding, therefore, 789 flood disaster records were also extracted from the entire disaster dataset to explore the relationship with the construction of masonry walls.

## Results

### Frequency and time division of masonry city wall construction

**Fig 1:**
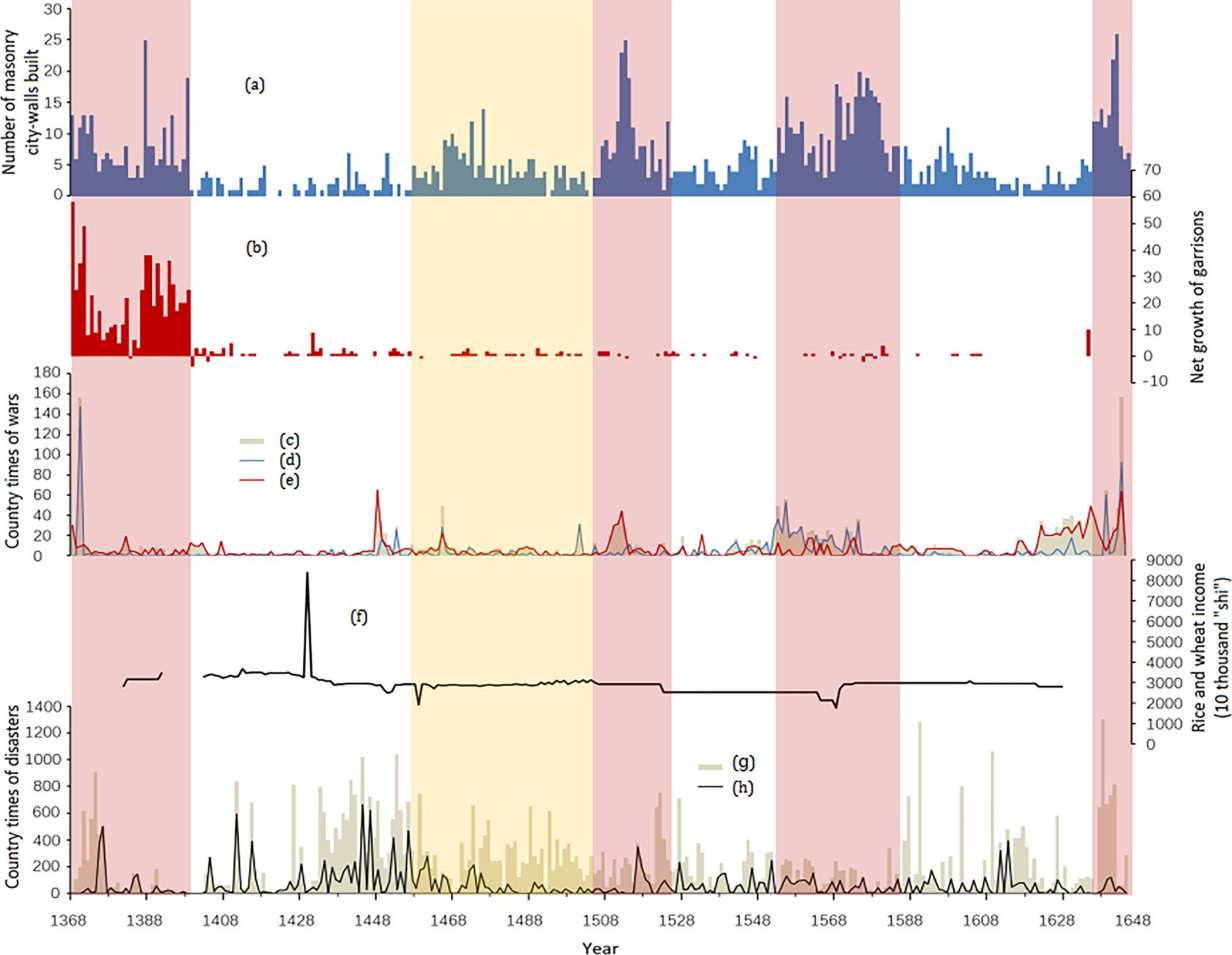
The masonry city walls construction, garrisons setting up and abandon, wars, rice and wheat revenue, and disasters sequences in the Ming Dynasty (1368–1644). (a) Masonry city walls construction sequence of the Ming Dynasty; (b) Garrisons setting up and abandon sequence; (c) Wars intensity sequence; (d) Foreign wars intensity sequence; (e) Internal wars intensity sequence; (f) Rice and wheat revenue of the central government sequence; (g) Disasters intensity sequence; (h) Flood intensity sequence. Note: “shi” was a unit of volume in ancient China and was commonly used to measure grain. 1 “shi”≈1.02 m^2^.

There were totally 1,618 masonry city walls built in China from the Eastern Han to the late Ming Dynasty; 125 walls were before the Ming Dynasty and 1,493 were in the Ming Dynasty. Fig 1(A) shows the changes in the frequency of masonry city wall construction in the Ming Dynasty. The periods of 1368–1398, 1506–1523, 1552–1585, and 1634–1644 had high-frequency construction activities of the masonry city walls (in red blocks, Fig 1) while 1399–1456, 1524–1551, 1586–1633 were with low-frequency (in blank blocks, Fig 1), and 1457–1505 was comparatively static (in yellow blocks, Fig 1).

We used the administrative classification method to categorize the administrative levels of the cities that built masonry walls [35]. Generally, cities that not directly governed the counties were labeled as “high-grade cities”, such as the cities of “Zhou” during the Northern and Southern Dynasties, the cities of “Dao” and “Lu” during the Tang and Song Dynasties, and the provincial capitals during the Yuan and Ming Dynasties. Cities that directly governed the counties were labeled as “county-governed cities”, such as the cities of “Jun” and “Zhou” during the Han, Sui and Tang Dynasties, the cities of “Fu” and “Zhou” during the Song, Yuan and Ming Dynasties. Cities that managed basic level of administrative districts were labeled as “county cities”. Coupled with the “self-owned land garrisons” and “no land garrisons” of the Ming Dynasty, all cities were divided into 5 categories. Table 1 shows the number, frequency, and types of masonry walls built from the Eastern Han to the late Ming Dynasty.

**Table 1:** The number, frequency, and types of the masonry city walls built from the Eastern Han to the late Ming Dynasty (25–1644 AD) in China.

Dynasties or periods	Time (AD)	Duration(Year)	Total number	Annual average	high-grade cities	govern counties cities	County cities	own land garrisons
Eastern Han	25–219	195	1	0.01	1	0	0	
South & North	220–589	370	3	0.01	2	1	0	
Sui & Tang	590–959	370	25	0.07	0	20	5	
Song	960–1279	320	51	0.16	0	35	16	
Yuan (early)	1280–1350	71	7	0.10	0	3	4	
Yuan (later)	1351–1367	17	38	2.24	3[2]	15[6]	20	
Ming	1368–1644	276	1493	5.41	11[11]	311[200]	969[97]	202
	1368–1398	31	248	8.00	7[7]	102[92]	38[27]	101
	1399–1456	58	88	1.52	1[1]	18[16]	30[14]	39
	1457–1505	49	210	4.29	0	32[20]	167[24]	11
	1506–1523	18	177	9.83	0	26[14]	150[3]	1
	1524–1551	28	119	4.25	0	19[7]	97[2]	3
	1552–1585	33	360	10.91	3[3]	67[34]	257[17]	33
	1586–1633	48	160	3.33	0	33[12]	114[9]	13
	1634–1644	11	131	11.91	0	14[5]	116[1]	1

Note: The numbers in square brackets indicate the number of masonry walls built in the local government cities where garrisons were stationed.

### Spatial distribution of masonry city wall construction in various periods

**Fig 2:**
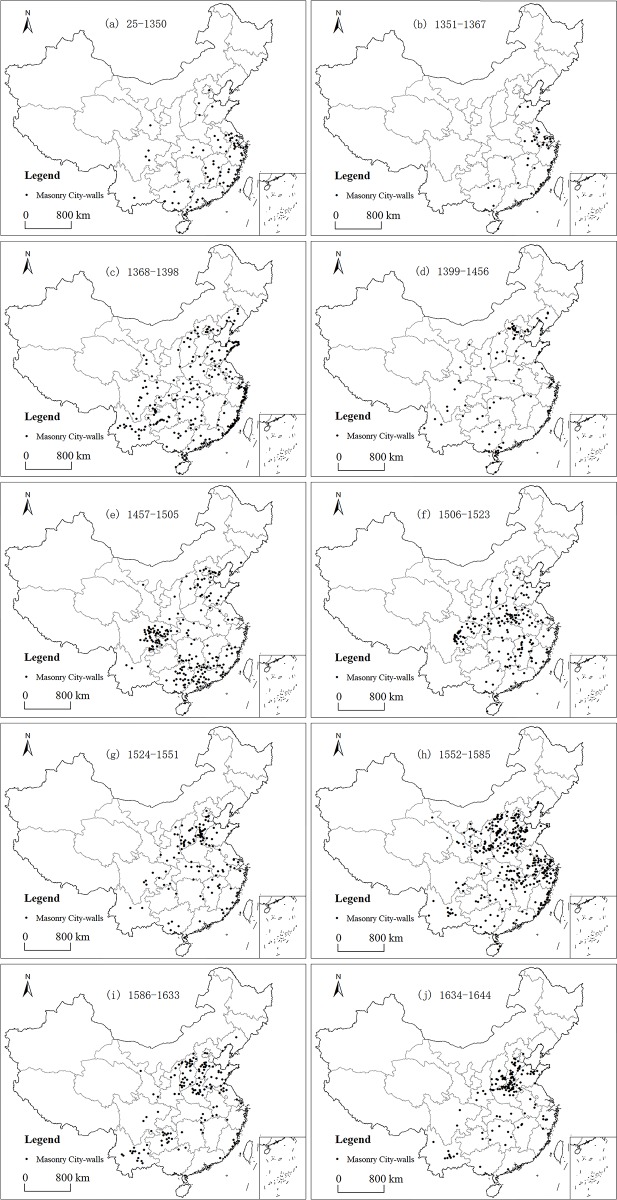
The distribution of the masonry city walls built from the Eastern Han to the late Ming Dynasty (25–1644 AD) in China.

We used ArcGIS to map the distribution of masonry city walls during various periods. This process resulted in 10 spatial distribution maps of masonry wall construction from the Eastern Han to the Ming Dynasty; we integrated the masonry walls during the Eastern Han and the early Yuan Dynasty into a single map considering their limited numbers (Fig 2).

The Eastern Han to the early Yuan Dynasty (25–1350, Fig 2A) was a long, budding period for the construction of masonry walls in China. The first masonry city wall in China was found in today’s Sichuan Province (Western China), however, the masonry walls in this period were concentrated in the southeast of China, including today’s Jiangsu, Zhejiang, Jiangxi, Fujian, and Guangdong Provinces, where 71.26% of these cities had higher administrative levels than the county level. The figure in Table 1 shows that construction of masonry walls during 25–1350 was mainly in the cities with higher administrative grade. Therefore, they had not been popularized in China during this period.

With the outbreak of war in the end of the Yuan Dynasty in 1351 AD, Zhu Yuanzhang, who later became the founding emperor of the Ming Dynasty, participated in the uprising and led to the collapse of the Yuan Dynasty in 1368. The 17 years’ turbulence became an independent historical unit and witnessed the loss of sovereignty of the central government in Beijing over the southern region of the Yuan Dynasty. As shown in Table 1 and Fig 2B, 38 masonry walls were built during this period and the annual average construction number was significantly higher than the previous period. The administrative grades of the construction cities were relatively average, but the geographical concentration trend in the Yangtze River Delta. Though the distribution range of masonry walls during this period was still very limited, it had gained considerable popularity in the Yangtze River Delta.

Fig 2C shows the construction of 248 masonry city walls in 1368–1398 (i.e. during the reign of Zhu Yuanzhang) with an unprecedented level of averagely 8 cities per year, which marked the beginning of history of the mass construction of masonry walls in the Ming Dynasty. Geographically, the construction of masonry walls in 1368–1398 broke through the southeast region and expanded to most regions controlled by the Ming Dynasty, and gathered along the coast and the major roads between Yunnan and Guizhou Provinces. Garrisons of this period played an important role in the construction of masonry walls. Of the cities built with masonry walls in 1368–1398, 126 cities were stationed with garrisons (50.81%), and 101 were self-owned land garrisons (40.73%), totally accounted for 91.53%. The sudden decrease in the annual number of construction in 1399–1456 did not alter the characteristics of geographical distribution and city types (see Fig 2D). For example, the construction activities then were still scattered throughout the country with a certain degree of cluster around Beijing indicating that the important role of the garrisons remained. For cities built with masonry walls in 1399–1456, 31 were stationed with garrisons (35.23%), and 39 were self-owned land garrisons (44.32%), totally accounted for 79.55%.

After 1457, the large-scale construction revived until the final collapse of the Ming Dynasty in 1644. Construction activities during this time could be divided into 6 periods, but were vastly different with previous periods. Table 1 shows the administrative level of the cities built with masonry walls after 1457. Among the 1,157 cities, 901 were county-level cities (92.98%) far exceeding the previous proportion. In addition, the influence of the garrison system was insignificant. Finally, these cities were no longer evenly distributed throughout the county. That is, there were several clustered areas and sparsely built areas during each period. In 1457–1505, the construction of masonry walls was concentrated in the Sichuan, Guangxi and Guangdong Provinces and the surrounding areas of Beijing (Fig 2E). In 1506–1523, the clustered areas were in Sichuan, Henan and Jiangxi Provinces, as well as southern Shaanxi and northern Anhui Provinces (Fig 2F). In 1524–1551, construction activities mainly occurred in the south of today’s Shanxi and Hebei Provinces (Fig 2G). In 1552–1585, mass construction appeared throughout North China and the middle and lower reaches of the Yangtze River, including Hebei, Henan, Shaanxi, Shanxi, Shandong, Jiangsu, Anhui, Zhejiang, Jiangxi, and Hubei Provinces (Fig 2H). In 1586–1633, mass construction activities in Hebei, Henan, Shanxi and Shandong Provinces continued, and accompanied with Yunnan and Guizhou Provinces (Fig 2I). Finally in 1634–1644, construction activities remained in four Provinces of Shanxi, Hebei, Henan, and Shandong (Fig 2J). The construction of masonry walls after 1457 were obviously regional and in phase; there were only sporadic construction activities in other non-concentrated areas.

## Discussion

### Masonry city walls were mainly distributed in southern China before the Ming Dynasty

The masonry city walls built between the Eastern Han Dynasty and the end of the Yuan Dynasty (25–1367) were mainly located in southern China (Fig 2A and 2B). According to previous research, a large number of city walls were built in the northern China during this long period, and the number often exceeds that of the south [26]. But they were mainly the pounded earth walls [7, 10–12, 16]11. China has a long history of building pounded earth city walls, dating back to the end of the Neolithic age [36]. Much of northern China was covered with loess, which becomes hard after pounded and was suitable for building walls [37]. Before the Song Dynasty (960–1279), the north was always the political and economic center of China. After the Song Dynasty, the economy of the north lagged behind that of the south, but it was still the political center. If necessary, the north was able to build many strong masonry walls, but it did not. Until to the end of the Yuan Dynasty (1352), the emperor ordered the building of city walls throughout the country [38], Hebei, Shanxi, Shaanxi, Jiangxi, Jiangsu and Zhejiang province responded positively and built many city walls [26]. But the north (Hebei, Shanxi, Shaanxi) still built earth walls primarily, and in the south (Jiangxi, Jiangsu, Zhejiang) masonry walls accounted for a large proportion. Comparing the situation of the construction of city walls in the south and the north, it could be considered that before the Ming Dynasty, there were two different methods of building in the north and the south of China: the traditional pounded earth city walls were adopted in the north, while the masonry walls with local characteristics popular in the south gradually.

This phenomenon was caused by the specific geographical environment and the rising political and economic status in south China before the Ming Dynasty. After the Southern and Northern Dynasties (220–589), southern China developed gradually and its political and economic status rose, resulting in a higher demand for urban defense than before. However, the environment in the south was not suitable for pounded earth walls like the north. People in the Tang and Song Dynasties (618–1279) noticed that the soil in the south was not suitable for pounded earth, and the walls had to be built of masonry. For example, in the Tang Dynasty, people thought the soil in E’zhou (Hubei Province) and Sichuan was so bad that bricks had to be used to build the walls [39]. People in the Song Dynasty believed that the earth walls in the south and the southeast were easy to collapse and needed to be built with masonry [40–42]. Zhu Xi (1130–1200), a famous scholar in the Southern Song Dynasty, also believed that the soil in the Central Plains (Henan Province) was dense, while that in the south was sparse, which would be easily damaged without masonry [43]. In addition, most cities in the south were built along rivers. The foundation of walls were easy to be eroded by water and needed to be protected with masonry. For example, Fuyang, Taizhou (Zhejiang Province), Jianchang [44], Xinghua [45] (Fujian Province), and Luzhou [40] (Sichuan Province), etc. in the Song Dynasty.

Masonry city walls were mainly distributed in the south, while earth walls were mainly used in north. This phenomenon was gradually formed under the influence of specific natural and human environment. Although it was true that the masonry wall was stronger than the earth wall, its appearance and popularity in southern China before the Ming Dynasty did not seem to be a deliberate result of the pursuit of some kind of stronger urban defense facilities beyond the earth walls. However, it should be an alternative measure when the traditional earth wall could not meet the need of urban defense. It was a strategy for the ancients to transform the method of construction city walls according to the characteristics of the natural environment.

### Factors determining the construction of masonry city walls in the Ming Dynasty

The peak period of the establishment of the garrisons was in 1369–1398 (Fig 1B). This period coincided perfectly with the peak period of the construction of masonry walls. Specifically, the number of established garrisons in 1368–1371 was the highest and gradually declined thereafter; it experienced a short trough during 1382–1385 and gradually recovered to the end of the peak period in 1398. The number of construction of masonry walls, at the same time, also experienced similar changes (Fig 1A). For example, after 1398, few new garrisons were set up, and the number of masonry walls built in 1399–1456 also decreased significantly (Table 1). Our results indicate that a certain correlation existed between the garrisons and masonry wall construction during 1368–1456.

Previous studies believed that the mass construction of masonry walls in the Ming Dynasty was related to the widespread use of firearms during the war [14–16]. However, our results indicate that this correlation was not obvious before 1398 (Fig 1C, 1D and 1E). Before 1456, less war appeared except the invasion of Japanese pirates in 1370, thus showing a negative correlation with the construction of masonry walls during the same period. The results may be attributable to the active and constructive motivation rather than the stimulation of wars. The war intensity reached a peak in 1448 (mainly due to internal warfare) while the construction of masonry walls also reached a small peak later. Since then, the construction of masonry walls had relatively corresponded with the sequence of war intensity. For example, three peaks of construction activities in 1506–1523, 1552–1585 and 1634–1644 corresponded with the three war intensity peaks. Geographically, the construction activities were clustered in the inland during the peaks of 1506–1523 and 1634–1644, which corresponded with the peaks of the internal wars. Similarly, with the peak of foreign wars in 1552–1585, the construction activities clustered in the North and Northwest China (against Mongolians), the middle and lower reaches of the Yangtze River, and the southeast coast (against Japanese pirates). The results suggest that the construction of masonry walls might be significantly associated with the war factor after 1457.

The economic factor might not have a strong correlation with the construction of masonry walls. As a proxy to economic factors, the income of rice and wheat of the central government had little fluctuations (except the abnormal value in 1429) and was generally stable at a level of approximately 30 million “shi” per year. (Fig 1F). Researchers found that except the border areas, the major funding source for the construction of the city walls in the inland of the Ming Dynasty came from local area [46]. The relatively stable fiscal revenue indicates that the government usually had a stable economic funding source for the expensive construction projects.

The construction activities of masonry walls were correlated with natural disasters during 1399–1644, but the correlation with floods was not significant (Fig 1G and 1H). Meanwhile, there was also a correlation between the disasters and the wars, especially for the internal wars. The correlation between disasters and the construction of masonry walls includes two aspects. First, disasters caused a series of economic problems such as food production reduction and famine, leading to the reduction of total economic output. This influence in turn spread to the government’s fiscal revenue, resulting in the demand reduction of mass construction projects. Therefore, the low incidence of masonry wall construction in 1399–1456 came with the high incidence of disasters in the same period. Second, floods could cause damage to the city wall buildings, but not serious enough to necessitate the construction of masonry walls. Construction of masonry walls normally appeared as one type of government measures to maintain social security when all kinds of disasters accumulated to a serious degree and caused social unrest. After 1457, the change patterns in the construction of masonry walls, wars, and disasters reflected situations aforementioned.

From the above analysis, it can be seen that the garrison system probably dominated the construction of masonry walls from 1368 to 1456, but its influence almost disappeared after that. The war factors had little influence in the early Ming Dynasty, but it almost determined the construction of masonry walls in 1457–1644. Economic and disaster factors were also related to the wall construction. These factors all had influences on the walls construction, but none of them could be regard as the dominant factor. The analysis of various factors deepened the understanding of the construction of masonry walls in the Ming Dynasty, but it was not enough to explain the whole event completely. It is necessary to go beyond the mono-causal or similarly simple explanations of causation.

### The construction of masonry walls was a phenomenon of cultural diffusion

Unlike traditional views, we were more inclined to see the mass construction of masonry walls in the Ming Dynasty as a large-scale cultural diffusion phenomenon. Before the Ming Dynasty, they were defense facilities with local characteristics and were mainly distributed in southern China. In the Ming Dynasty, they spread dramatically throughout the country. The construction of masonry walls was mainly an act of governmental. The Ming Dynasty could be defined as a mandatory system with territorial rights and an orderly flow of information, resources, and people to ensure the security of the dynasty. The central government headed by the emperor dominated the communication system. Official institutions underneath constituted the channels for the circulation of information, resources and people [47]. In this case, the diffusion process of masonry walls reflected the complex interaction between the central government and the local governments in the Ming Dynasty.

Central government played a decisive role in the origin (1368–1456) of this cultural diffusion. The masonry walls built during this period had a common spatial distribution with no obvious clustered areas; the type of these cities was dominated by the cities with higher administrative grade and garrisons. These features were clearly demonstrated inherent strong power to support and intervene; this power was the central government of the Ming Dynasty. This was closely related to the activities of the Ming army at that time. The predecessor of the Ming army was the Red Turbans army led by Zhu Yuanzhang at the end of the Yuan Dynasty. Zhu joined the Red Turbans in 1352, and by 1368, Zhu and his troops had fought in the Yangtze River Delta for 16 years. The Yangtze River Delta was where the masonry walls were most densely distributed before the Ming Dynasty. The terrain was flat and not conducive to defence. In this environment, the city wall was an important support to seek refuge and destroy the enemies. Wars tended to focus on several important cities. It was in this situation that Zhu Yuanzhang’s army built a large number of masonry walls. When Zhu’s army captured important cities, they usually built masonry walls immediately, and most of the works were organized by the generals who defended the cities. For example, in 1352 in Anqing (Anhui Province) [48], and later in Jiangyin, Changzhou (Jiangsu Province) [49], Anji, Changxing (Zhejiang Province) [48], etc. Even Zhu himself organized such works (Hezhou, Anhui Province) [48]. When Zhu Yuanzhang conquered the Yangtze River Delta in 1368 and marched across China, the Ming army maintained the practice of building masonry walls in important cities. Most of the construction were organized by local military officers or higher-ranking generals. For example, general Hua Yunlong built city walls near Beiping (Beijing) in 1370, Fu Youde in Sichuan and Yunnan Province in 1383, Tang He in Zhejiang Province in 1386, and Lan Yu in Sichuan in 1389 [48], etc. We did not find any orders (if documented) from the central government of the Ming Dynasty to build masonry walls throughout the country, but the masonry walls met the demand of the government’s construction of the garrison system during that period. Therefore, the implementation and development of the national system expedited the spread of masonry walls throughout the country. The support of the central government ensured the breadth of the cultural diffusion.

On the other hand, local governments were faced with completely different situations from the central government. They were not only subject to the vertical administration of superior institutions, but were also affected by their embedded regional social networks. The process of the diffusion of masonry city walls in county cities during 1457–1644 suggests that the geographical structure of the defense facilities in cities at this level was different with the high-grade cities during the previous period. The construction activities of masonry walls in county-level cities were mainly clustered in areas and times that were affected by external factors (e.g., wars and natural disasters); Central-to-local government plans no longer existed. Many historical documents clearly stated that the masonry walls were built during in this period in response to the threat of wars. For example, Guangdong, the cluster region in 1457–1505, it was recorded that masonry walls were built in response to the riots of ethnic minorities [48]; In 1506–1523, Henan and Jiangxi built masonry walls on a large scale to deal with bandits and refugees [48, 50–51]; In 1552–1585 it was concentrated in the middle and lower reaches of the Yangtze River to deal with the threat of Japanese pirates [52–53]. The diffusion of masonry walls at the county level reflected the recognition and depth by the local community.

However, the diffusion of masonry walls in county cities in 1457–1644 may not simply be explained by the external factors (e.g., wars and natural disasters). Rather, the key explanation lies in understanding the relations between the central government and local community in ancient China. Simply put, it was not a matched relationship between rule and obedience, command and execution, but an interactive state [54]. In addition to the strong intervention mechanism from the central government, to tackle with the external factors, the local communities were more likely to choose economically effective means within a limited regional social network. Once the approach was proved to be effective, it would be learnt and labeled as a created culture by the local community, and gradually be assimilated into its cultural traditions [55]. In the middle and late Ming Dynasty, the significance of the garrisons as a military system existed in nominal terms [56]. When the security and stability of the local community was affected by external factors (e.g., wars and natural disasters), the involved local governments and regional social networks would respond by obtaining effective resources. In this case, the available solid masonry walls in the high-grade cities became a reasonable choice for the local community. With the popularity of the masonry walls among the high-grade cities and garrisons countrywide before 1456, the construction activities were no longer planned by the central government. A consistent effect was achieved based on used the masonry walls as the same reference objects in these area, resulting in the deeper diffusion of these defense facilities. The planning of the central government, the interaction between local community and the central government, and the stimulation of external factors had contributed to the diffusion of masonry city walls in the Ming Dynasty.

## Conclusion

The first masonry city wall in China appeared in the 1^st^ century AD, which was far behind the civilizations of the Mesopotamia, the ancient India, and the ancient Greek-Roman civilization. However, the emergence of masonry walls in China did not make it popular. From the 1^st^ century to the first half of the 14^th^ century, the total number of the construction of masonry walls was only 125 with an annual average number below 0.1. The masonry walls were mainly scattered in the south of China. In the middle of the 14^th^ century, the outbreak of war in the end of the Yuan Dynasty witnessed a leading mass construction of masonry walls in the Yangtze River Delta. After the establishment of the Ming Dynasty in 1368, masonry walls began to sprawl dramatically in China. In the Ming Dynasty, a total of 1,493 masonry walls were built and the average annual number reached to 5.41. Following this trend, by the middle of the 17^th^ century, most Chinese cities had set up masonry city walls as their defense facilities. The construction activities of 1368–1456 were all over the country, but specifically targeted to high-grade administrative cities and garrisons. The rising construction activities were associated with the establishment of the garrison system, as a result of the planned implementation of the central government. The construction activities of 1457–1644 appeared in the corresponding cluster areas during different periods, and normally the cities were at county level. The garrison system failed during this period, so the construction activities were stimulated by external factors (e.g., wars and disasters). This result could be explained by the matched areas and periods of the wars and disasters. In summary, instead of exploring specific reasons for the mass construction of the masonry walls in the Ming Dynasty, we attribute it to a cultural diffusion phenomenon. The central government’s planning, the complex interactions between local government and community, and the stimulation of external factors (e.g., wars and disasters) had commonly contributed to the popularity of masonry city walls in the Ming Dynasty.

Based on the collection and analysis of historical documents, we restored the spatio-temporal process of the construction of masonry walls from the 1st century to the mid-17th century in China, and selected the potential factors (i.e., wars, garrison settings, economy, and natural disasters) to conduct time series analysis. In the era of the pre-industrial revolution, the large-scale diffusion of an object in human civilization was a very interesting process. Exploring this process is significant for understanding the development and migration of human civilization. We attempt to use relatively simple elements (e.g., influencing factors) to seek for one of these diffusion phenomena. Our study serves as a glance at the development of masonry walls in Chinese history, however, there is still much room to explore in the future.

## Supporting information

S1 Shapefile This script collects information from the references on the construction of masonry city walls in China during 1^st^ to 17^th^ centuries AD, and compiles them into a shapefile. (ZIP)

S1 Table This script creates the figures used in text. (XLSX)
